# Simultaneous in vivo PET/MRI using fluorine-18 labeled Fe_3_O_4_@Al(OH)_3_ nanoparticles: comparison of nanoparticle and nanoparticle-labeled stem cell distribution

**DOI:** 10.1186/s13550-020-00655-9

**Published:** 2020-06-30

**Authors:** Sarah Belderbos, Manuel Antonio González-Gómez, Frederik Cleeren, Jens Wouters, Yolanda Piñeiro, Christophe M. Deroose, An Coosemans, Willy Gsell, Guy Bormans, Jose Rivas, Uwe Himmelreich

**Affiliations:** 1 Biomedical MRI, Department of Imaging and Pathology, KU Leuven, 3000 Leuven, Belgium; 2 NANOMAG Group, Department of Applied Physics, Technological Research Institute, Universidade de Santiago de Compostela, 15782 Santiago de Compostela, Spain; 3 Radiopharmaceutical Research, Department of Pharmaceutical and Pharmacological Sciences, KU Leuven, 3000 Leuven, Belgium; 4 Molecular Small Animal Imaging Center (MoSAIC), KU Leuven, 3000 Leuven, Belgium; 5 Nuclear Medicine and Molecular Imaging, Department of Imaging and Pathology, KU Leuven/UZ Leuven, 3000 Leuven, Belgium; 6 Laboratory for Tumor Immunology and Immunotherapy, ImmunOvar Research Group, Department of Oncology, Leuven Cancer Institute, KU Leuven, 3000 Leuven, Belgium; 7 Department of Gynaecology and Obstetrics, UZ Leuven, 3000 Leuven, Belgium

**Keywords:** Cell tracking, Nanoparticles, Preclinical imaging, Radiolabeling, PET/MRI, Stem cells

## Abstract

**Background:**

Mesenchymal stem cells (MSCs) have shown potential for treatment of different diseases. However, their working mechanism is still unknown. To elucidate this, the non-invasive and longitudinal tracking of MSCs would be beneficial. Both iron oxide-based nanoparticles (Fe_3_O_4_ NPs) for magnetic resonance imaging (MRI) and radiotracers for positron emission tomography (PET) have shown potential as in vivo cell imaging agents. However, they are limited by their negative contrast and lack of spatial information as well as short half-life, respectively. In this proof-of-principle study, we evaluated the potential of Fe_3_O_4_@Al(OH)_3_ NPs as dual PET/MRI contrast agents, as they allow stable binding of [^18^F]F^−^ ions to the NPs and thus, NP visualization and quantification with both imaging modalities.

**Results:**

^18^F-labeled Fe_3_O_4_@Al(OH)_3_ NPs (radiolabeled NPs) or mouse MSCs (mMSCs) labeled with these radiolabeled NPs were intravenously injected in healthy C57Bl/6 mice, and their biodistribution was studied using simultaneous PET/MRI acquisition. While liver uptake of radiolabeled NPs was seen with both PET and MRI, mMSCs uptake in the lungs could only be observed with PET. Even some initial loss of fluoride label did not impair NPs/mMSCs visualization. Furthermore, no negative effects on blood cell populations were seen after injection of either the NPs or mMSCs, indicating good biocompatibility.

**Conclusion:**

We present the application of novel ^18^F-labeled Fe_3_O_4_@Al(OH)_3_ NPs as safe cell tracking agents for simultaneous PET/MRI. Combining both modalities allows fast and easy NP and mMSC localization and quantification using PET at early time points, while MRI provides high-resolution, anatomic background information and long-term NP follow-up, hereby overcoming limitations of the individual imaging modalities.

## Background

Mesenchymal stem cells (MSCs) are multipotent stromal cells that have been shown to have high potential for disease treatment, e.g., Crohn’s disease, ocular disorders, spinal cord injuries, and cartilage repair [1–5]. Furthermore, MSCs have been shown to target tumors and their microenvironment, and potentially play a role in tumor cell therapy [6, 7]. However, their in vivo behavior and role in the different therapeutic areas is still poorly understood [6, 8, 9]. The non-invasive visualization of MSCs could help to elucidate this working mechanism in the various applications. To do so, different in vivo cell tracking strategies have been applied, including the indirect labeling of MSCs with viral vectors [10–12]. While this allows long-term detection of the cells, spatial resolution is often poor, and genetic modification of the MSCs is necessary, hereby, limiting the clinical applicability of this strategy [13].

A second approach includes direct labeling of MSCs using either nanoparticles (NPs) or radiotracers for visualization with magnetic resonance imaging (MRI) or positron emission tomography (PET), respectively. Superparamagnetic iron oxide (Fe_3_O_4_) NPs have been used extensively due to low toxicity, high sensitivity, and their potential as MRI cell tracking agents [11, 14, 15]. Furthermore, these NPs can be engineered to be excellent drug delivery vehicles, retaining them until their release is triggered at the site of interest [16, 17]. However, their hypointense contrast is often difficult to visualize, interpret, and quantify throughout the body, especially in regions with hypointense background signal like the lungs or blood vessels [18, 19].

On the other hand, several PET radiotracers have also shown their potential as cell tracking agents [20–23]. While their detection is highly sensitive and specific, the lack of anatomical information and low spatial resolution can make the exact localization of the compounds/cells difficult [24]. Furthermore, the limited tracer half-life precludes long-term cell visualization after pre-labeling [13], restricting their use as long-term cell tracking agents. Therefore, the development of novel contrast agents overcoming both limitations is necessary.

Overcoming disadvantages of single modality imaging may also require the use of dual contrast agents in multimodal approaches, e.g., simultaneously acquired PET/MRI. Combining PET and MRI not only provides anatomical images with great soft tissue contrast (MRI), but also highly sensitive and specific functional, cellular (PET and MRI), and molecular images with low background (PET) depending on the radiotracer used [25, 26]. While the time period that a tracer can be followed in vivo using PET is restricted and depends on the radionuclide’s half-life, MRI allows long-term detection of dual contrast agents. Furthermore, simultaneous PET/MRI acquisition facilitates the image co-registration of both modalities [26].

Several approaches were already established for the development of novel dual PET/MRI contrast agents by coupling MRI contrast agents, e.g., Fe_3_O_4_ nanoparticles, liposomes, or gadolinium, with different radioactive isotopes, e.g., fluorine-18 (^18^F), galium-68, copper-64, iodine-124, or zirconium-89, to allow their visualization with both PET and MRI [27–32]. Compared to other commonly used PET isotopes, the use of ^18^F is preferred as it is easy and cheap in production and leads to high resolution PET images due to abundant low-energy positron emission [33]. Furthermore, ^18^F-metal complexes can easily be formed, with one of the strongest and most stable bonds with Al(OH)_3_ [33, 34].

Recently, we have developed, synthesized, and extensively characterized the physico-chemical properties of novel ^18^F-labeled Fe_3_O_4_@Al(OH)_3_ NPs for simultaneous PET/MR imaging [35]. Labeling of the nanostructures with [^18^F]NaF was rapid and relatively stable in media representing physiological conditions. Furthermore, we showed that both the uptake of these non-toxic RL NPs by mouse MSCs (mMSCs) and the visualization of the RL NPs and RL NP-labeled mMSCs in agar phantoms using both PET and MRI were feasible.

The encouraging results from our in vitro work led us to investigate the ^18^F-labeled Fe_3_O_4_@Al(OH)_3_ NPs as in vivo contrast agents. The aim of the present study was to evaluate the potential of these novel nanostructures as mMSC cell tracking agents for simultaneous PET/MRI after their intravenous (IV) injection in healthy mice, and to confirm the results using ex vivo measurements. Hereby, we hope to combine the advantages of MRI-based cell tracking in terms of long-lasting contrast and high spatial resolution with those of PET-based cell tracking in terms of sensitivity and contrast specificity.

## Materials and methods

### Radiolabeling of Fe_3_O_4_@Al(OH)_3_ NPs and cell labeling

[^18^F]NaF was produced as described before [35]. All experiments were performed with a final concentration of 0.6–2.1 GBq [^18^F]NaF/mL (on average 1.0 GBq/mL). Full experimental details on the tracer production and a description of the NPs can be found in the Supplementary Materials and Methods.

Radiolabeling of Fe_3_O_4_@Al(OH)_3_ NPs was performed based on the protocol described by Cui et al. and further optimized in our previous study [31, 35]. In brief, NPs containing 1.70 ± 0.1 mM iron were incubated with 3–10 MBq of [^18^F]NaF for 10 min at room temperature. Afterwards, they were centrifuged for 20 min at 4000 rpm and resuspended in saline. NPs labeled with 3 MBq [^18^F]NaF were injected IV via the tail vein of the mice, while those labeled with 10 MBq [^18^F]NaF were used for mMSCs labeling.

mMSCs were obtained from the laboratory of Stem Cell Biology and Embryology at KU Leuven. They were transduced with a lentiviral vector encoding for firefly luciferase (fLuc) and the enhanced green fluorescent protein in our lab [11, 36], and cultured and labeled with NPs as previously described [35]. More experimental details can also be found in the Supplementary Materials and Methods. After labeling, 1 × 10^5^ (RL) NP-labeled mMSCs were resuspended in saline for IV injection.

### Animals

Animal experiments described in this manuscript were approved by the Ethical Committee of KU Leuven (ECD p259/2015) and were conducted according to Belgian (Royal Decree, 29 May 2013), Flemish (Decision of the Flemish Government to adapt the Royal Decree of 29 May 2013, 17 February 2017), and European (Directive 2010/63/EU) regulations on the protection of animals used for scientific purposes.

Six to eight-week old wild-type C57Bl/6 mice were housed in individually ventilated cages with ad libitum access to food and water. Mice were randomly assigned to the different groups (see below). All imaging, blood sampling procedures, and IV injections were performed under anesthesia using 2–3% isoflurane (IsoVET, 100 mg/g, Eurovet, Piramal Critical Care, West Drayton, UK) in 100% O_2_.

**Fig. 1:**
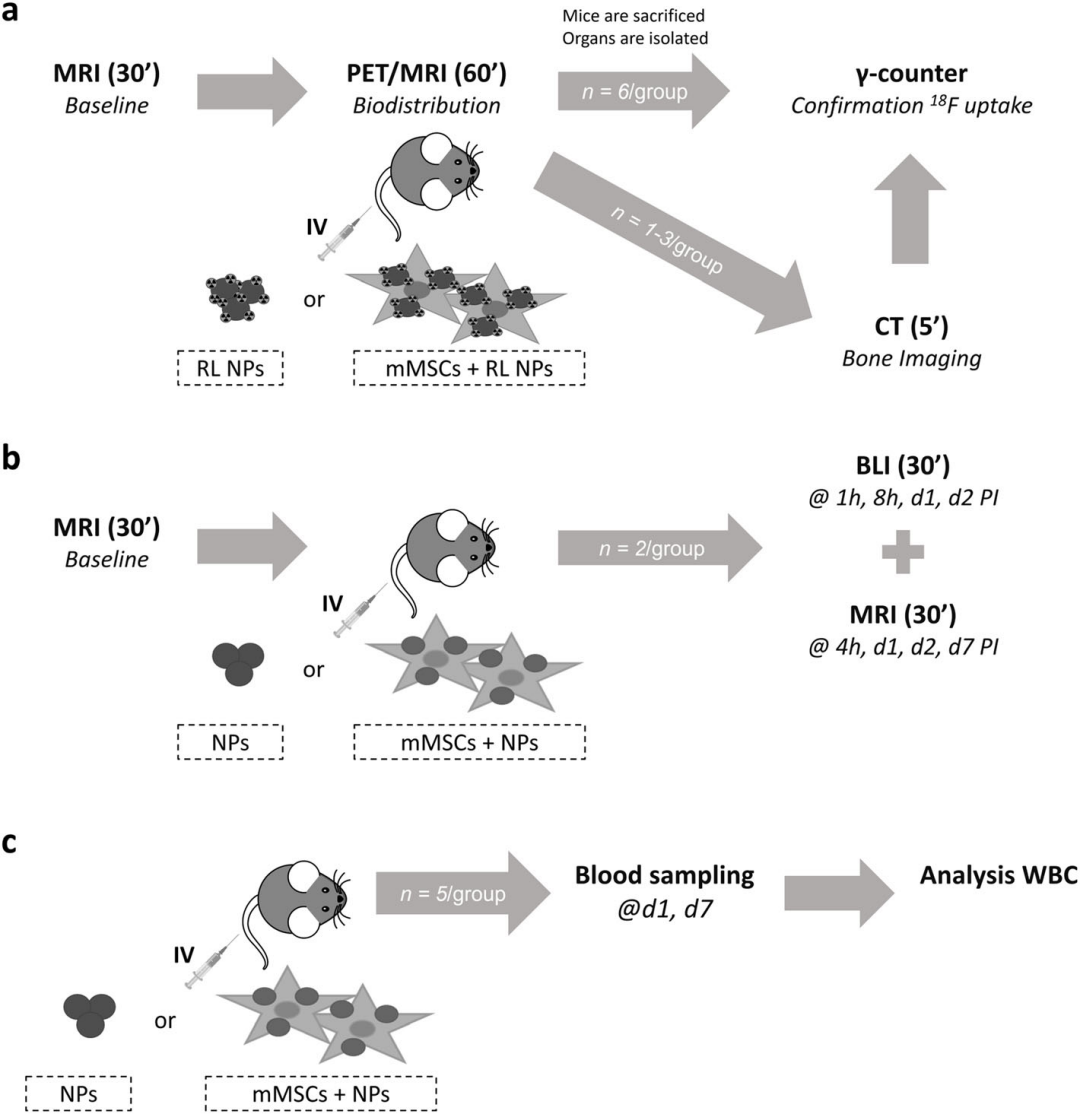
Experimental overview. **a** After acquisition of baseline MRI scans, ^18^F-labeled Fe_3_O_4_@Al(OH)_3_ nanoparticles (radiolabeled; RL NPs) or mouse mesenchymal stem cells (mMSCs) labeled with such NPs (mMSCs + RL NPs) are injected intravenously (IV) during simultaneous PET/MRI acquisition to follow-up their respective short-term biodistribution over time. In the control group, saline or free [^18^F]NaF was injected. Afterwards, a small cohort of mice underwent CT imaging to visualize uptake in bones. Finally, animals were sacrificed; different organs were isolated and analyzed using the gamma-counter. **b** Biodistribution of non-RL (cold) NPs or mMSCs labeled with them was studied over a period of 7 days post-injection (PI) using both MRI and bioluminescence imaging (BLI) after the IV injection of the NPs/cells. **c** After IV injection of cold Fe_3_O_4_@Al(OH)_3_ nanoparticles or mMSCs labeled with them, blood samples were taken on day one and day seven PI to analyze the white blood cell (WBC) population. Control groups in **b** and **c** were injected with saline

Mice were injected via the lateral tail vein with either 100 μL saline containing (a) RL (*n =* 8) or (b) cold Fe_3_O_4_@Al(OH)_3_ NPs with an iron content of 1.6 mM iron (*n =* 7), (c, d) 1 × 10^5^ mMSCs labeled with such NPs (cold, *n =* 7 or RL, *n =* 7) in experimental groups or 100 μL (e) saline (*n =* 14) or (f) [^18^F]NaF (*n =* 3) in control groups. While short-term biodistribution studies were performed in mice injected with RL NPs/RL NP-labeled mMSCs, other experiments were completed on animals injected with cold NPs or NP-labeled mMSCs. An experimental outline can be found in Fig. 1. In all cases, injections were performed using an in-house made catheter consisting of a 30-gauge needle connected to polyurethane tubing (inner diameter of 0.015 in.; SAI Infusion Technologies, Lake Villa, IL, USA). The catheter was filled with a sterile 0.9% saline solution containing 1/10,000 heparin (Heparine LEO®, Pharma A/S, 5.000 I.E./mL, LEO Pharma A/S, Ballerup, Denmark) before injection of the experimental compound in the mouse’s tail vein. Afterwards, the catheter was flushed with a small volume of 0.9% saline to ensure that the full experimental volume was injected into the mice.

At the end of the study, animals were sacrificed using an intraperitoneal injection of 100 μL of sodium pentobarbital (Dolethal®; Vetoquinol, Northamptonshire, UK) and exsanguinated once reflexes were not present anymore. The lungs were inflated with 0.7 mL formalin (10% in PBS (Sigma-Aldrich); ChemLab, Zedelgem, Belgium) using a 22-gauge catheter via the trachea before excision. Afterwards, the liver, lungs, and spleen were isolated, and ^18^F activity in the liver, lungs, and spleen was counted with a 2480 Wizard^2^ Automatic Gamma Counter (20 s protocol; PerkinElmer, Waltham, MA, USA). To calculate the standardized uptake values (SUV) within each organ, the following formula was applied:

1 $$ \mathrm{SUV}=\raisebox{1ex}{$\frac{\mathrm{counts}\ \mathrm{per}\ \mathrm{minute}\ \left(\mathrm{CPM}\right)\ \mathrm{in}\ \mathrm{organ}}{\mathrm{weight}\ \mathrm{of}\ \mathrm{organ}\ \left(\mathrm{g}\right)}$}\!\left/ \!\raisebox{-1ex}{$\frac{\mathrm{CPM}\ \mathrm{in}\ \mathrm{body}}{\mathrm{weight}\ \mathrm{of}\ \mathrm{animal}\ \left(\mathrm{g}\right)}$}\right. $$

Total counts per minute (CPM) in body was calculated by decay-correcting the injected dose to the time of sacrifice (approximately 10 min after last scan) and converting this activity into CPM using the MBq to CPM conversion factor for the gamma-counter.

### In vivo follow-up of biodistribution of Fe_3_O_4_@Al(OH)_3_ NPs and NP-labeled mMSCs

#### Short-term follow-up

##### PET/MRI

All images were acquired using a BioSpec 70/30 small animal MRI scanner equipped with a silicon photomultiplier PET insert (Bruker Biospin, Ettlingen, Germany). Further details on hardware and experimental protocols/parameters can be found in Table 1 and the Supplementary Materials and Methods. To study NP/cell biodistribution, 100 μL saline containing RL NPs (0.91 ± 0.18 MBq [^18^F]F^−^ upon injection) or 10^5^ RL NP-labeled mMSCs (0.23 ± 0.08 MBq [^18^F]F^−^ upon injection) were injected via the tail vein of the mice, 1 min after the start of the simultaneous PET/MRI acquisition (1 h static PET scan and a 20 min dynamic contrast enhanced (DCE) MRI scan in combination with additional MRI scans (see below)). In control groups, 100 µL saline or [^18^F]NaF (1.15 ± 0.18 MBq) was injected. DCE-MRI was acquired after local field homogeneity adjustments. Furthermore, a 3D T_2_-weighted anatomical MR scan and a T_2_ map covering a large part of the liver and part of the lungs were acquired before and after injection of NPs/mMSCs.

**Table 1:** Overview of MRI sequences and scan parameters

	3D T_**2**_-weighted	3D DCE	T_**2**_ map
Sequence	RARE	FLASH	MSME
Effective echo time	26 ms	4 ms	8–64 ms with 8 ms increments
Repetition time	500 ms	20 ms	1800 ms
Rare factor	8	-	-
Averages	1	1	2
Number of repetitions	1	20	1
Flip angle	-	30°	-
Frame period	-	60.33 s	-
Matrix	150 × 350 × 128	128 × 256 × 45	192 × 128
Field of view	35 mm × 80 mm × 30 mm	35 mm × 80 mm × 30 mm	42 mm × 30 mm
Slices	-	-	Axial slice orientation 2 intercalated slice packages 8 slices of 1 mm in each package 1 mm slice gap
Respiration-gated	No	No	Yes
Scan time	13.5 min	20.07 min	11.52 min + time for gating

*3D* Three dimensional, *DCE* Dynamic contrast enhanced, *RARE* Rapid acquisition with refocused echoes, *FLASH* Fast low angle shot, *MSME* Multi slice multi echo

##### Computed tomography

After acquisition of the PET/MRI scans, the mouse bed was moved to a dedicated small animal CT scanner (SkyScan 1278, Bruker CT, Kontich, Belgium) for a subgroup of animals, and a whole-body CT scan was acquired (parameters see Supplementary Materials and Methods).

#### Long-term follow-up

For long-term follow-up of the distribution of cold NPs or NP-labeled mMSCs, both MRI and bioluminescence imaging (BLI) were performed for up to 7 days. Experimental details can be found in the Supplementary Materials and Methods.

### Image analysis

Average T_2_ values of the liver were determined after generation of parametric T_2_ maps using the manufacturer’s Paravision 6.0.1. software (Bruker Biospin). DCE images were analyzed using an in-house written tool and 3D Slicer version 4.8.1. to determine the mean liver signal intensity.

CT data was reconstructed and analyzed with Bruker CT software (Nrecon version 1.16.10.4. and CT An version 1.16.3.0.) in combination with ImageJ to obtain a CT-based bone mask.

The manufacturer’s Albira software (Bruker Biospin) was used to reconstruct the acquired static PET scans using the maximum likelihood estimation method (12 iterations, 0.5 mm isotropic resolution). The scans were reframed into 28 frames, which were dynamically reconstructed using the same algorithm. All PET images were analyzed using PMOD version 3.9 (PMOD Technologies, Zürich, Switzerland). These were manually overlaid with the 3D T_2_-weighted MRI or the CT-based bone mask to determine the mean standardized uptake values (SUV) in the liver, lungs, spleen, kidneys, and bladder, or to quantify the mean SUV of the bone structure, respectively, within the PMOD FuseIT tool. Furthermore, a spherical volume of interest with a 1 mm radius was placed inside the left ventricle of the heart to determine the clearance rate from the blood pool. Maximum intensity projection (MIP) images were created based on the average of the last six acquired frames (30 min).

To analyze BLI data, we made use of the manufacturer’s Living Image software, version 4.5.2. (Perkin Elmer). A rectangular region of interest (width = 1.86 cm, height = 1.59 cm) was drawn around the lungs. The total flux within this area was quantified.

More information on the different image analysis procedures can be found in the Supplementary Materials and Methods.

### Evaluation of leukocytosis

To evaluate the effect of NPs or NP-labeled mMSCs on leukocytosis, mice were injected IV with cold NPs or NP-labeled cells. Blood was taken from the animals, and the number and differentiation of white blood cells (WBC) was analyzed. For further details, see Supplementary Materials and Methods.

### Statistical analysis

All statistical analyses were performed in GraphPad Prism version 8.0.2 (GraphPad Software Inc., San Diego, CA, USA). A two-tailed *p* value was considered statistically significant when *p* < 0.05. Comparisons between groups, between groups at different time points or between different experimental groups and organs, were studied using a one-way analysis of variance (ANOVA) with Bonferroni correction for multiple comparisons, a repeated measures two-way ANOVA with Bonferroni correction for multiple comparisons or a two-way ANOVA with Bonferroni correction for multiple comparisons, respectively. In case of missing values, data were analyzed by means of a mixed-effects model with Bonferroni correction. In all figures, data are represented as mean ± standard deviation.

## Results

### In vivo and ex vivo measurements reveal uptake of ^18^F-labeled NPs in the liver, while RL NP-labeled mMSCs are taken up in the lungs

**Fig. 2:**
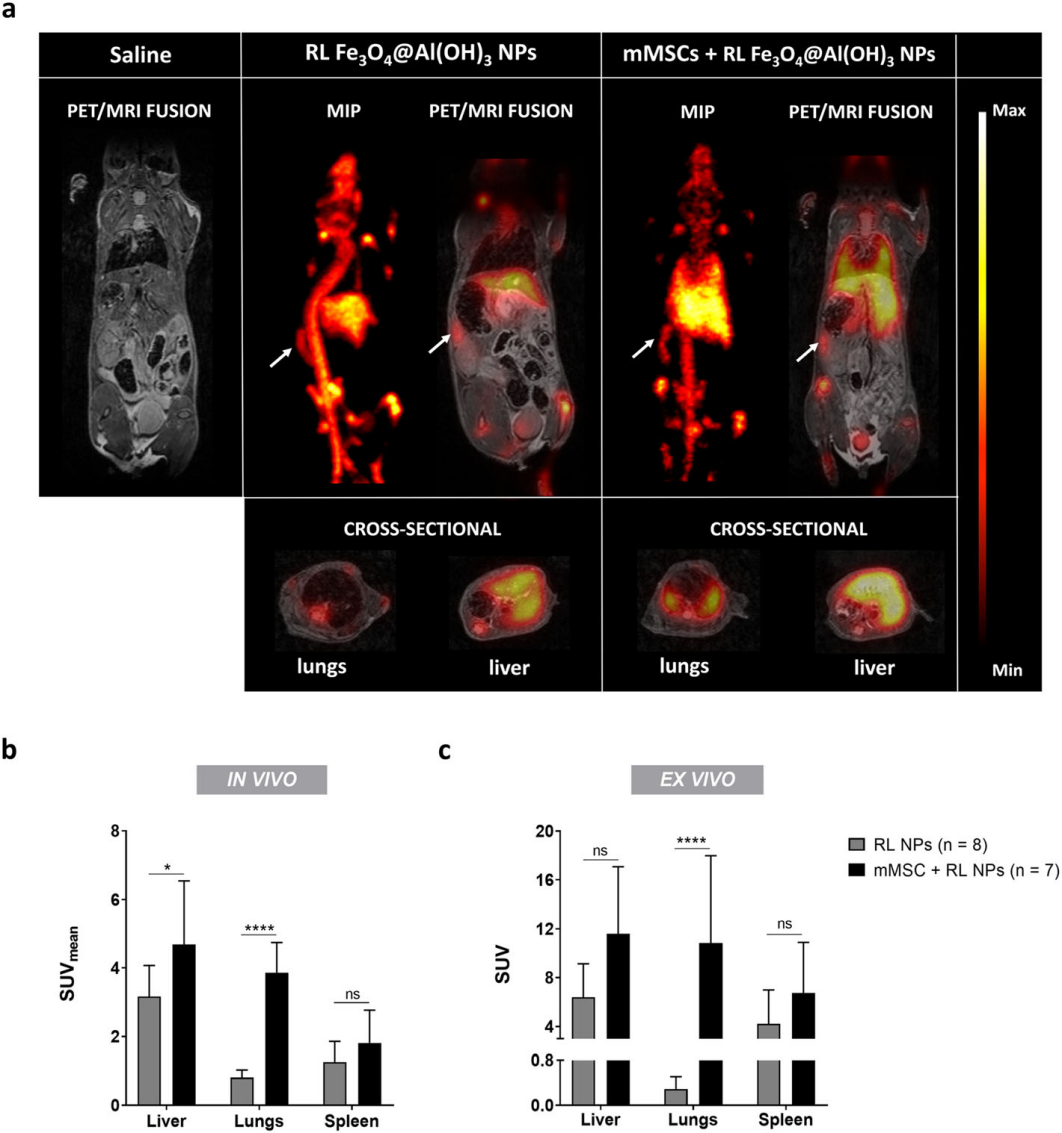
In and ex vivo biodistribution of ^18^F-labeled Fe_3_O_4_@Al(OH)_3_ NPs or mMSCs labeled with these NPs. Mice were injected intravenously with saline, ^18^F-labeled Fe_3_O_4_@Al(OH)_3_ NPs (radiolabeled or RL NPs), or 100,000 mouse mesenchymal stem cells (mMSCs) labeled with RL NPs (mMSCs + RL NPs) while simultaneous PET/MRI images were acquired. **a** Representative maximum intensity projection (MIP) images and overlays of T_2_-weighted anatomical MR images and ^18^F-PET images (SUV = 0–9) show the uptake of ^18^F-labeled Fe_3_O_4_@Al(OH)_3_ NPs in the liver, while mMSCs labeled with RL NPs mainly accumulate in the lungs. In the latter group, signal from free NPs was also detected in the liver. Furthermore, uptake of ^18^F-labeled Fe_3_O_4_@Al(OH)_3_ NPs can be appreciated in the spleen of both groups, as indicated by the white arrows. **b**, **c** Corresponding quantification of **b** in vivo and **c** ex vivo standardized uptake values (SUV) of the liver, lungs, and spleen confirm the images. In vivo values represent the average SUV over the 1 h scan time. Indicated statistics are based on two-way ANOVA between the different groups with Bonferroni’s multiple comparisons test (*p < 0.05, ****p < 0.0001, ns, not significant)

Mice were injected with ^18^F-labeled-Fe_3_O_4_@Al(OH)_3_ NPs or RL NP-labeled mMSCs, and scanned using simultaneous PET/MRI to study the in vivo biodistribution of both the NPs and the NP-labeled cells. Based on the PET signal intensities, the overlaid 1 h static PET and anatomical MRI scans show uptake of the RL NPs in mainly the liver, while RL NP-labeled mMSCs accumulate both in the lungs and liver (Fig. 2a). Both liver and lung uptake of RL NP-labeled mMSCs was significantly higher than for RL NPs (*p* = 0.0177 and *p* < 0.0001, respectively) (Fig. 2b). A SUV_liver/lung_ ratio of 4 and 1.2 was found for the groups injected with RL NPs and RL NP-labeled mMSCs, respectively. In both experimental groups relatively high spleen uptake was observed. However, the difference between both experimental groups was not significant (*p* = 0.8798) (Fig. 2a, b).

To confirm uptake of ^18^F-labeled NPs in the liver, lungs, and spleen, mice were sacrificed and organs were isolated for subsequent gamma-counting*.* Ex vivo measurements confirm the in vivo data, showing higher uptake in the liver, even though not significant (*p* = 0.0687), and in the lungs (*p* < 0.0001) of mice injected with RL NP-labeled mMSCs, while similar uptake in the spleen of both groups was noted (*p* = 0.7595) (Fig. 2c). Differences by a factor of 2 to 3.7 were found between in vivo and ex vivo measurements for the liver, lungs, and spleen.

### Immediate and stable uptake of RL NPs and RL NP-labeled mMSCs in the liver and lungs, respectively, is seen on ^18^F-PET scans

Dynamic reframing of PET scans into 28 frames (20 of 1 min to match DCE-MRI and eight frames of 5 min for later time points) was performed to study the biodistribution of NPs/cells in the liver and lungs over the first hour and to determine the image-derived left ventricle input functions.

**Fig. 3:**
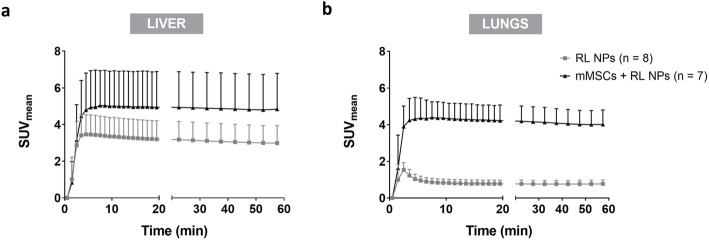
Dynamic uptake pattern of ^18^F-labeled-Fe_3_O_4_@Al(OH)_3_ NPs or mMSCs labeled with these NPs. The static scans were reframed into 20 frames of 1 min and 8 frames of 5 min to study the uptake pattern of radiolabeled (RL) NPs and mMSCs over the time period of acquisition within **a** the liver and **b** the lungs. Uptake of RL NPs in the liver can be seen in both experimental groups as seen by the increase in mean standardized uptake value (SUV_mean_), with a slow decreasing trend at later time points. On the other hand, the first pass of RL NPs in the lungs can be visualized, while there is stable uptake of mMSCs after 2 to 3 min

Radiotracer uptake was seen in the liver immediately after injection in both experimental groups (Fig. 3a). No significant differences were found between the maximum ^18^F uptake and later time points, even though a decreasing trend was seen. Furthermore, no differences were detected between both groups.

Stable accumulation of RL NP-labeled mMSCs was also seen in the lungs of these mice. Similarly, to the decrease in the liver at later time points, a decreasing trend in the lungs was noted. In contrast, rapid first-pass of RL NPs was seen in the lungs. Furthermore, a significant difference in the lungs between the RL NPs and RL NP-labeled mMSCs group was found (*p* < 0.0001 from frame 3 onwards; Fig. 3b).

Rapid blood pool clearance of both the RL NPs and RL NPs-labeled mMSCs was seen after their intravenous injection, resembling the blood clearance of the free tracer (Fig. S1). A significant difference right after injection was only measured between each of these experimental groups and [^18^F]NaF-injected animals, while no other differences were seen at any other time point.

### MRI confirms the uptake of Fe_3_O_4_@Al(OH)_3_ NPs in the liver, while mMSCs do not survive in the lungs as proven by BLI

**Fig. 4:**
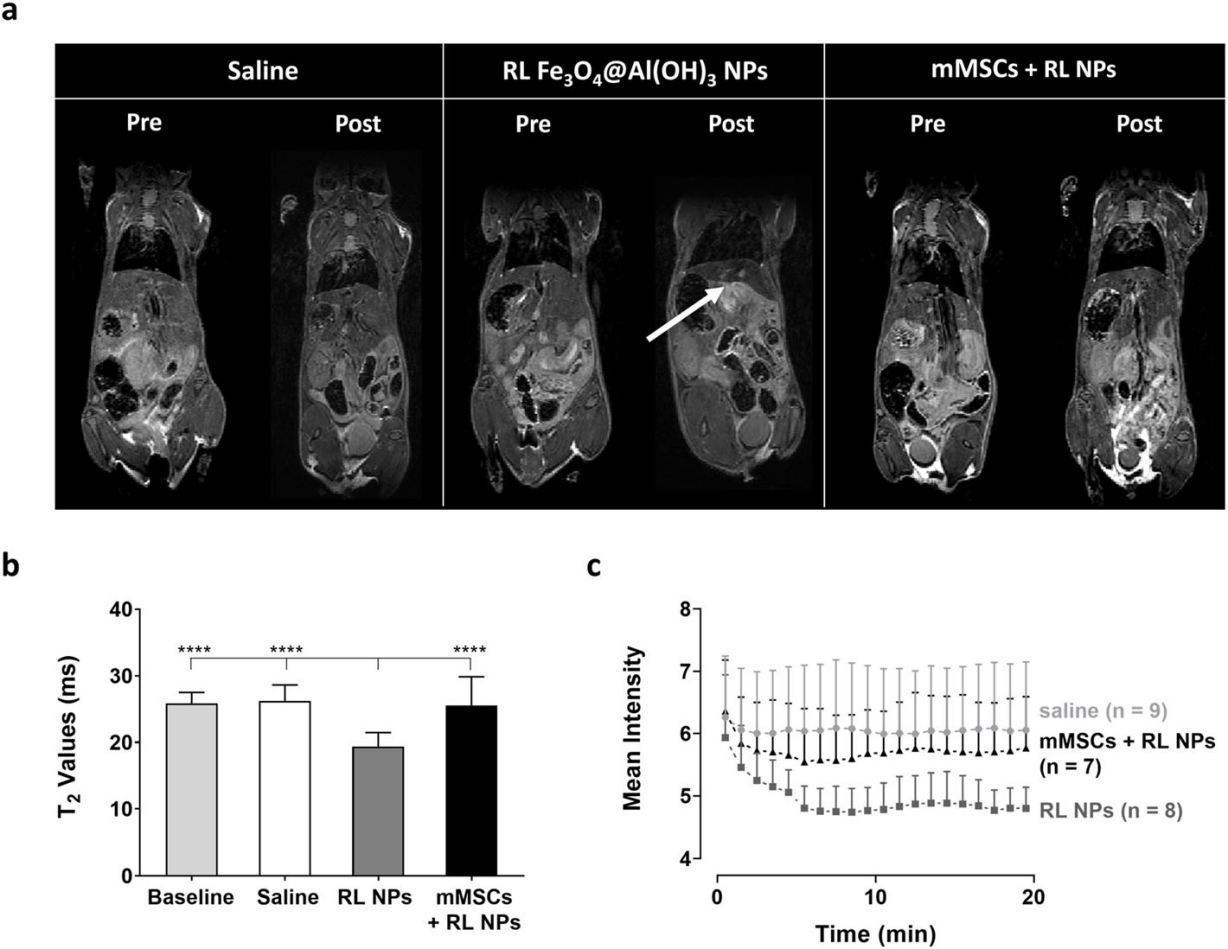
Whole-body MRI for localization of ^18^F-labeled Fe_3_O_4_@Al(OH)_3_ NPs or mMSCs labeled with these NPs. A dynamic contrast enhanced (DCE) MRI, 3D T_2_-weighted anatomical MRI, and T2 map were acquired to study the biodistribution of ^18^F-labeled Fe_3_O_4_@Al(OH)_3_ NPs (RL NPs) and mMSCs labeled with RL NPs (mMSCs + RL NPs) in comparison with the control group (injection of saline). **a** A characteristic hypointense liver can be seen on the 3D T_2_-weighted anatomical scans after the injection of the RL NPs as indicated by the white arrow. **b** The significant decrease in T_2_ values of the liver after injection of the RL NPs in comparison to baseline, the saline control and the mMSC + RL NPs group confirms the uptake of the NPs in the liver (one-way ANOVA with Bonferroni correction for multiple testing; ****p < 0. 0001). **c** DCE-MRI further confirms the uptake of RL NPs by the significant decrease in mean signal intensity in the liver 5 min after injection (*p* < 0.05 when compared to baseline; two-way repeated-measures ANOVA with Bonferroni correction)

To evaluate the fate of the NPs or NP-labeled mMSCs over a seven-day time period, mice were injected with them and scanned repeatedly using MRI and BLI to track the NPs and viable mMSC, respectively (Fig. 4 and Fig. S2).

Within 1 h, RL NP-injected mice presented with a hypointense signal in the liver on 3D T_2_-weighted anatomical MRI scans in combination with a significant decrease in T_2_ values in the liver (*p* < 0.001) when compared to baseline, saline-injected mice, and mice injected with NP-labeled mMSCs (Fig. 4a, b), indicative of NP uptake. Furthermore, these measurements correspond to the steepest decrease in mean signal intensity that was seen on DCE-MRI after injection of RL NPs. This was significant in comparison to saline-injected animals starting 5 min after NP injection (*p* < 0.05). While the uptake of NPs in the form of a decrease in signal intensity is also seen after injection of RL NP-labeled mMSCs, significant differences compared to the control group are only observed 9 to 10 min after injection of mMSCs (*p* < 0.05; Fig. 4c).

In a longitudinal follow-up experiment, a hypointense signal from the liver and accompanying decrease in T_2_ values were seen from 4 h post injection (PI) of Fe_3_O_4_@Al(OH)_3_ NPs onwards, demonstrating their uptake. An increase in T_2_ values was seen 7 days PI, suggesting NP clearance from the liver. On the other hand, after injection of NP-labeled mMSCs, hypointense contrast of the organ, and thus NP uptake was less pronounced. This was confirmed by a smaller decrease in T_2_ values of the liver (Fig. S2a, b).

Furthermore, BLI showed the presence of viable mMSCs in the lungs until 24 h PI, while no bioluminescence signal could be detected in any other organ or in the control group after NP injection (Fig. S2c). Quantification of total flux reveals large intra-group variability right after cell injection (1 h PI). However, in all animals a sudden decrease in light intensity in the lungs was seen after 8 h with a decrease to background signal within 48 h PI (Fig. S2d). This decrease in BLI signal was accompanied by a further decrease of T_2_ values of the liver. Combined these results indicate that mMSCs death in the lungs is associated with NP release and clearance by the reticuloendothelial system, which can be monitored by a decrease in T_2_ values of the liver in MR images.

### In vivo, some ^18^F dissociates from the NPs

As small, but detectable PET signal from the bladder and different bone areas was observed on PET/MRI in both experimental groups (Fig. 2a), we suspected that part of the ^18^F-label dissociates from the NPs after injection into the mice. Therefore, CT scans were acquired from a subgroup of mice to visualize their bone structures. A CT-based bone mask was manually overlaid with the ^18^F-PET images to determine ^18^F bone uptake.

**Fig. 5:**
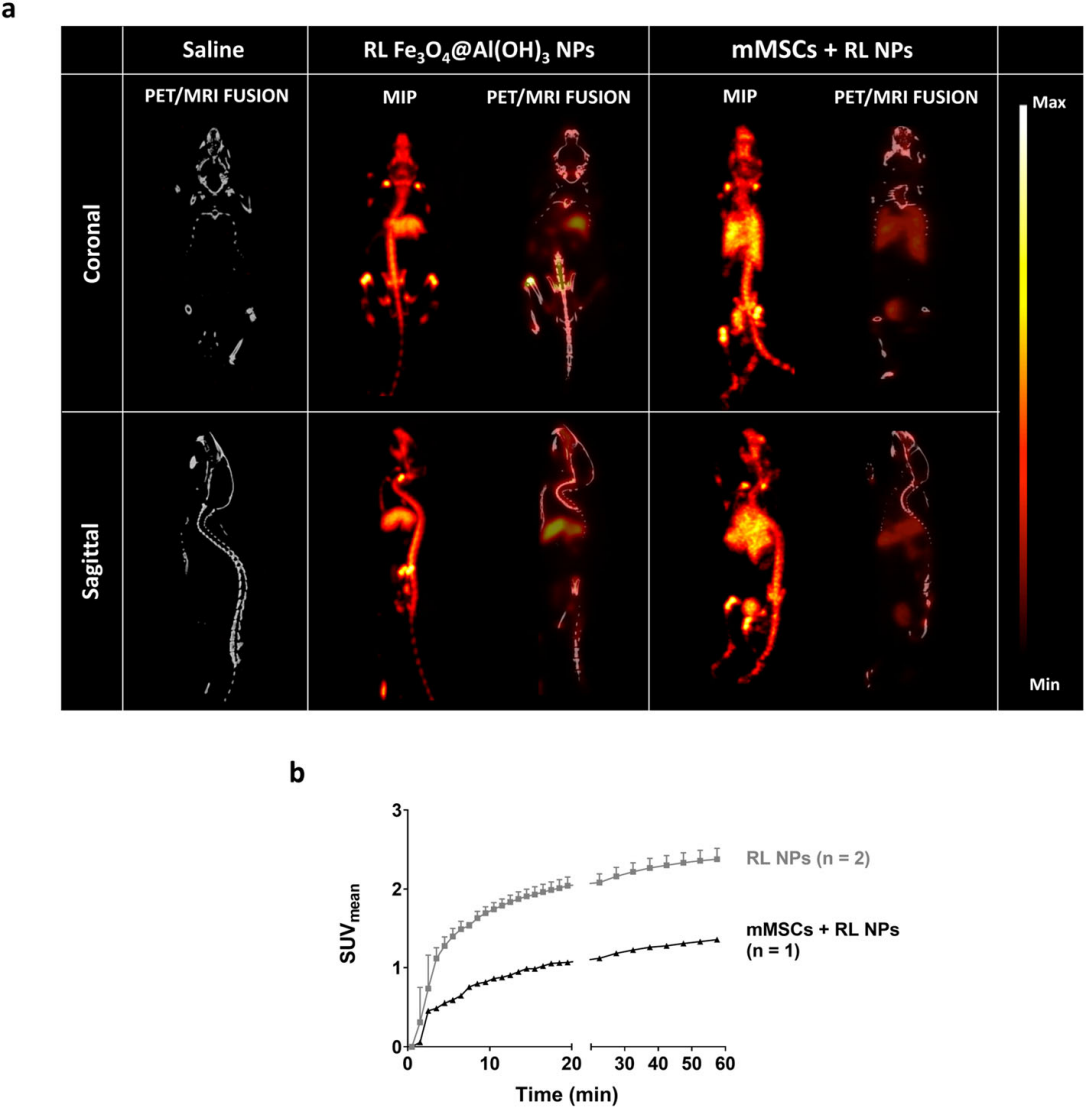
Bone uptake after injection of ^18^F-labeled Fe_3_O_4_@Al(OH)_3_ NPs or mMSCs labeled with these NPs. After acquisition of the last PET/MRI scan, a CT scan was acquired for a subgroup of mice to visualize the bone structure. **a** After injection of either RL NPs or mMSCs labeled with RL NPs (mMSCs + RL NPs), ^18^F uptake was visualized in the bones of the mice on the maximum intensity projection (MIP) images and overlays of CT-based bone mask and 1 h static ^18^F-PET (SUV = 0–9). **b** Corresponding quantification (mean standardized uptake values, SUV_mean_) confirms the gradual uptake in bone. Significant differences were found between both groups starting from 4 min post injection (PI) of the tracer (frame 5) based on a repeated measures two-way ANOVA with Bonferroni correction for multiple comparisons (4 min PI: *p* < 0.05, 5–11 min PI: *p* < 0.01, from 12 min PI on: *p* < 0.001)

The overlays reveal some tracer uptake into the skeleton of the mice after injection of both RL NPs and RL NPs-labeled mMSCs (Fig. 5a). The SUV_liver_/SUV_bone_ ratio was 1.82 and 2.35, for mice injected with RL NPs or RL NPs-labeled mMSCs, respectively, while the SUV_lungs_/SUV_bone_ ratio for the latter group was 2.58. Furthermore, quantification of the dynamic ^18^F-signal indicates gradual dissociation of the radioactive label from the NPs in both groups, with less free ^18^F present after injection of radiolabeled cells (Fig. 5b).

As a control group, mice were injected with [^18^F]NaF to determine the tracer uptake in the bone of the mice. Most ^18^F uptake was seen in all bones across the body, similarly to the experimental groups. This was significantly higher when compared to the uptake in the liver or lungs (Fig. S3a, b). Furthermore, the bone uptake was substantially higher after [^18^F]NaF injection when compared to RL NPs or RL NP-labeled mMSCs with SUV_liver_/SUV_bone_ ratio of 0.15 and the SUV_lungs_/SUV_bone_ ratio of 0.26 for the [^18^F]NaF injection. This ^18^F uptake was rapid and almost stable 25 min after injection (Fig. S3c). Lastly, a significantly higher ^18^F uptake in the bladder was also measured after injection of the free tracer compared to both other groups, while no significant differences in kidney uptake were seen (Fig. S4).

### Injection of NPs or NP-labeled mMSCs has no effect on leukocytosis

As previous studies have shown that NPs can have negative effects on the immune system of mice [37, 38], we studied the effects of the Fe_3_O_4_@Al(OH)_3_ NP and labeled mMSCs in vivo. On day one and seven PI, blood samples were analyzed for the number of WBC. No differences in the total number WBCs or differentiated subgroups, i.e., lymphocytes, monocytes, and granulocytes, were found between the different groups on day one or seven or between the different time points (Fig. S5). Last, no significant decrease in the weight of the animals was seen after the injection of either the NPs or mMSCs labeled with NPs when compared to baseline (data not shown).

## Discussion

The recent emergence of preclinical and clinical PET/MRI scanners has generated new perspectives for improved, dual PET/MRI contrast agents, overcoming disadvantages of those used for single modality imaging. In a previous study, we have reported on the production, characterization, and the fast in vitro radiolabeling of Fe_3_O_4_@Al(OH)_3_ NPs with [^18^F]F^−^ [35]. Furthermore, using a PET/MRI scanner for simultaneous data acquisition, we were able to directly compare PET and MRI contrast of ^18^F-labeled Fe_3_O_4_@Al(OH)_3_ NPs and mMSCs labeled with them. Here, we tested the potential of ^18^F-labeled Fe_3_O_4_@Al(OH)_3_ NPs as a contrast agent for in vivo cell tracking applications in mice using simultaneously acquired PET/MRI.

To conjugate an ^18^F-label to NPs, different radiofluorination strategies are available, including the use of conventional prosthetic groups, chelator-based radiolabeling using the Al^18^F-method or by click chemistry [39–42]. The latter could be used in a pretargeting approach, where NPs are injected before radiotracer administration, allowing imaging at later time points [42, 43]. However, all approaches require the derivatization of the NPs with a chelator or production of ^18^F-labeled prosthetic groups, which is more complex than the direct adsorption of [^18^F]F^−^ to the NPs. This approach is straightforward, but results in ^18^F-labeled constructs with potentially limited stability. Our method allowed low activity (less than 1 MBq) injections of ^18^F-labeled Fe_3_O_4_@Al(OH)_3_ NPs containing lower amounts of iron (11.85 μg), compared to previous studies that used NPs containing 60 μg of iron and up to 37 MBq of activity [31, 44, 45]. Despite this low concentration of iron and radioactivity level, using a highly sensitive PET insert and optimized MRI sequences, we were able to visualize at least 10^5^ cells both by PET and MRI compared to 8 million cells labeled with 0.555 MBq in earlier in vivo studies [46, 47]. For iron oxide-based NP cell labeling approaches, it is difficult to determine a minimum number of detectable cells as not only the R_2_ relaxivity of the labeling agent is important but also the background (T_2_ value) of the target organ/tissue. While the liver with its short T_2_ values (dark background) is not the most favorable organ for MRI-based cell imaging, other groups have suggested the possibility of single cell MR imaging against a homogeneous, bright background like the brain [48, 49]. In our experience, several hundred to a thousand cells are detectable by MRI in vivo [50–52].

After IV injection, blood clearance of both RL NPs and mMSCs labeled with these NPs was relatively fast. The slight delay in peak SUV after injection of either experimental compound can be explained by the short reframing time at the start of the scans and a minimal difference in injection rate, as both NPs and cells are manually injected. To minimize this variance, the same operator injected all animals. Furthermore, RL NPs were successfully visualized in the liver, most likely due to their uptake by Kupffer cells [53]. Both PET, i.e., high SUV_mean_, and MRI, i.e., hypointense contrast on anatomical MRI, decreased T_2_ values and decrease in mean signal intensity on DCE-MRI, were successful in detecting NP uptake in the liver. On the other hand, PET showed clear uptake of RL NP-labeled mMSCs in the liver and lungs. As previously reported, a large proportion of the (RL) NP-labeled mMSCs accumulated in the lungs—most likely being trapped in lung capillaries. These results were confirmed by BLI, which indicated the presences of viable cells in the lungs until 24 h PI, confirming earlier research by Eggenhofer and colleagues [54].

After injection of RL NP-labeled mMSCs, mice presented with a high contrast in the liver on both PET and MR images. This is most likely caused by macrophages that phagocytized dead or dying mMSCs, the presence of mMSCs debris or the presence of free NPs due to their release from the dying cells [9, 54, 55]. Conversely, a hypointense contrast on 3D T_2_-weighted anatomical MRI and decrease in T_2_ values of the liver were only seen from 4 h PI onwards, indicating that circulating NPs are not released immediately after injection. Only DCE-MRI of the liver showed a small, non-significant, decrease in signal intensity within 20 min after RL NP-labeled mMSCs injection. Interestingly, dynamic PET and MRI (DCE) data of NP accumulation in the liver are comparable, with similar time to maximum/minimum signal intensities in both groups.

The absence of contrast changes on MRI techniques in the liver and lungs right after injection of the mMSCs can be explained by reaching the detection limits of MR imaging. In the lungs, the low intrinsic MRI signal (close to noise level) renders the NPs detection impossible unless non-Cartesian sampling of the k-space is used to reach very short echo times (TE; ultra-short TE, zero TE) [56, 57]. As expected, conventional spin-echo (RARE and MSME) and gradient-echo (FLASH) sequences failed to identify the accumulation of the NPs in the lungs after cell injection.

Our study illustrates the advantages of combining PET and MRI for cell tracking. PET provides a quantitative read-out with high sensitivity and high specificity, independent of indirect parameters like MRI relaxation times. On the other hand, MRI provides the possibility of high spatial resolution, good soft tissue contrast for localization of NP/cell accumulation, and the possibility for longitudinal follow-up. Hereby, the limitations of PET in terms of limited tracer half-lives and low spatial resolution and the complications of MRI in terms of unfavorable background signal for some organs (i.e., lungs) and indirect quantification can be overcome.

Both experimental groups also present with spleen uptake, confirming NP clearance from the blood via the reticuloendothelial system [53]. Furthermore, some activity accumulated in the bones and bladder, indicating the release of free ^18^F [58]. Quantification of serial PET/CT scans reveals gradual tracer uptake in the mice’s skeleton, confirming the presence of free ^18^F. This could be explained by loss of the ^18^F-labeled aluminum hydroxide shell from the NPs or gradual release of the radiolabel from the NPs [31, 33, 59]. Noticeably, bone uptake was much higher after injection of free RL NPs compared to RL NP-labeled mMSCs, indicating a more stable trapping of the fluorine-18 label inside the cells. This is also in line with our previous in vitro experiments that indicated release of fluoride ions from NPs after exposure to serum-containing medium [35]. Furthermore, ^18^F bone uptake in both experimental groups was lower than in the control group injected with free [^18^F]NaF.

To validate our in vivo results, mice were sacrificed after the last scan and activity in the lungs, liver, and spleen were determined using a gamma-counter. While a similar relative trend was seen between the in vivo PET data and ex vivo gamma-counter data, a factor 2 to 3.7 difference in the absolute quantification was observed. Furthermore, a large difference between in vivo and ex vivo SUV_liver_/SUV_lungs_ ratio was detected. These differences can be explained by the partial volume effect (spill-over) from neighboring organs (i.e., liver to lungs after injection RL NPs and lungs to liver after injection of RL NP-labeled mMSCs) and photon attenuation in vivo [60, 61]. This is in particular pronounced for the experimental group that has received RL NPs as no trapping in the lungs was expected and seen ex vivo. Moreover, while PET values were calculated per organ volume, ex vivo biodistribution SUVs were determined per organ weight. Therefore, the density of different organs, mainly the lungs, also plays a role in the divergence between in vivo and ex vivo SUV values.

While semi-quantitative cell tracking was demonstrated in our PET/MRI study, caution must be taken. To the best of our knowledge, no kinetic modeling studies have been published for PET-based cell tracking. This is most likely due to the complexity of cell labeling studies. Instead of a simple two-compartment model, considering only an intracellular and extracellular compartment, one has to consider the gradual release of tracer from the NPs, NP stability, cell death, uptake of NPs by macrophages, and more. This was not only illustrated by the bone uptake in our study but also by a more careful consideration of the dynamic PET data of the liver. While one would expect a gradual SUV increase or steady state levels in the liver with clearance of NPs from circulation, we also found a gradual decrease after approximately 400–600 s in the RL NPs and RL NPs-labeled mMSCs groups, respectively, indicating loss of label.

Next to reliable quantification and new kinetic modeling methods, the absence of adverse side effects is necessary for future in vivo applications of Fe_3_O_4_@Al(OH)_3_ nanostructures. While the lack of toxicity in cell culture at the concentrations used in this study has been demonstrated before [35], the effect of the NPs/labeled cells on WBC was evaluated in vivo in immunocompetent mice. With the relatively low NP concentrations used in our study, no differences could be found in the total number of leukocytes or differentiated WBCs, in contrast to previous studies where adverse and/or inflammatory effects against both NPs containing Fe_3_O_4_ or aluminum and mesenchymal stem cells were found in vitro and in vivo [9, 37, 38, 62]. The presence of a polyacrylic acid coating on the NPs and the use of autologous mMSCs within this study do reduce the chance of an immune reaction against these novel NPs and the NP-labeled cells [20, 35, 36].

## Conclusion

We present the successful in vivo tracking of ^18^F-labeled-Fe_3_O_4_@Al(OH)_3_ NPs and RL NP-labeled mMSCs using simultaneously acquired PET/MRI. Large amounts of injected NPs were observed in the liver, while cells were visualized in the lungs. Even though, some ^18^F-label detached from the NPs, this did not have a negative effect on the visualization of either NPs or cells in vivo. Furthermore, no negative effects on the immune system were detected. Combining both imaging modalities clearly improves the visualization and quantification of the NPs/cells at early time points and furthermore, builds on the advantages of both techniques. After optimization of the radiolabeling strategy, these NPs present as promising candidates for further development into dual PET/MRI contrast agents for cell tracking applications.

## Supplementary information

**Additional file 1.** Supplementary information

## Data Availability

The datasets used and/or analyzed during the current study are available from the corresponding author on reasonable request.

## Abbreviations

ANOVA: Analysis of variance
3D: Three dimensional
Al(OH)_3_: Aluminum hydroxide
BLI: Bioluminescence imaging
CPM: Counts per minute
CT: Computed tomography
DCE: Dynamic contrast enhanced
Fe_3_O_4_: Iron oxide
Fe_3_O_4_@Al(OH)_3_: Iron oxide embedded in aluminum hydroxide
FLASH: Fast low angle shot
IV: Intravenous
(m)MSCs: (Mouse) mesenchymal stem cells
MRI: Magnetic resonance imaging
MSME: Multi slice multi echo
NP: Nanoparticle
PI: Post injection
RARE: Rapid acquisition with refocused echoes
RL: Radiolabeled
SUV: Standardized uptake value
TE: Echo time
WBC: White blood cells
