# Supplementary material for: Simultaneous in vivo PET/MRI using fluorine-18 labeled Fe3O4@Al(OH)3 nanoparticles: comparison of nanoparticle and nanoparticle-labeled stem cell distribution
Source: EJNMMI Res. 2020 Jun 30;10:73. doi: 10.1186/s13550-020-00655-9 (PMC7326875; doi:10.1186/s13550-020-00655-9)
Supplement: Supplementary file 1 — Additional file 1. Supplementary information [file 13550_2020_655_MOESM1_ESM.docx]

**Simultaneous in vivo PET/MRI using fluorine-18 labeled Fe_3_O_4_@Al(OH)_3_ nanoparticles: comparison of nanoparticle and nanoparticle labeled stem cell distribution**

**SUPPLEMENTARY INFORMATION**

Sarah Belderbos^1*^, Manuel Antonio González-Gómez^2*^, Frederik Cleeren^3^, Jens Wouters^4^, Yolanda Piñeiro^2^, Christophe M. Deroose^5^, An Coosemans^6,7^, Willy Gsell^1^, Guy Bormans^3^, Jose Rivas^2^, Uwe Himmelreich^1^

^1^ Biomedical MRI, Department of Imaging and Pathology, KU Leuven, 3000 Leuven, Belgium

^2^ NANOMAG Group, Department of Applied Physics, Technological Research Institute, Universidade de Santiago de Compostela, 15782 Santiago de Compostela, Spain

^3^ Radiopharmaceutical Research, Department of Pharmaceutical and Pharmacological Sciences, KU Leuven, 3000 Leuven, Belgium

^4^ Molecular Small Animal Imaging Center (MoSAIC), KU Leuven, Leuven, 3000 Leuven, Belgium

^5^ Nuclear Medicine and Molecular Imaging, Department of Imaging and Pathology, KU Leuven/UZ Leuven, 3000 Leuven, Belgium

^6^ Laboratory for Tumor Immunology and Immunotherapy, ImmunOvar Research Group, Department of Oncology, Leuven Cancer Institute, KU Leuven, 3000 Leuven, Belgium

^7^ Department of Gynaecology and Obstetrics, UZ Leuven, 3000, Leuven, Belgium

^*^ These authors contributed equally.

**Supplementary materials and methods**

- [^18^F]NaF production
- Cell labeling using Fe_3_O_4_@Al(OH)_3_ NPs
- In vivo follow-up of biodistribution of Fe_3_O_4_@Al(OH)_3_ NPs and NP-labeled mMSCs
- Image analysis
- Leukocytosis

**Supplementary figures**

- **Fig. S1.** Image-derived left ventricle input functions
- **Fig. S2.** Long-term follow-up of Fe_3_O_4_@Al(OH)_3_ NPs and mMSCs + NPs biodistribution
- **Fig. S3.** PET/MRI in combination with PET/CT after injection of [^18^F]NaF
- **Fig.** **S4.** Renal and bladder secretion of ^18^F
- **Fig. S5.** Evaluation of the effect on white blood cells after injection of Fe_3_O_4_@Al(OH)_3_ NPs or mMSCs labeled with these NPs

**Supplementary materials and methods**

***[^18^F]NaF production***

Fluorine-18 was produced as described in [1]. Production was done on site using a cyclotron (IBA Cyclone 18/9, IBA, Louvain-la-Neuve, Belgium) by irradiation of H_2_^18^O with 18-MeV protons.

[^18^F]F^−^ was separated from [^18^O]H_2_O by trapping on a Sep-Pak Light Accell plus QMA anion exchange cartridge (Cl^−^ form; Waters, Zellik, Belgium). The cartridge was washed with water (3 mL, HPCE grade; Sigma-Aldrich, Overijse, Belgium) and [^18^F]F^−^ was eluted from the cartridge with an aqueous solution of sodium chloride 0.9% (Mini-Plasco, solution for injection, B. Braun, Diegem, Belgium) to obtain a concentration of 0.6 – 2.1 GBq/mL (average: 1.0 GBq/mL) ready for further use.

***Cell labeling using Fe_3_O_4_@Al(OH)_3_ NPs***

Fe_3_O_4_@Al(OH)_3_ NPs with a hydrodynamic diameter of 573.2 ± 106.7 nm, a zeta potential of 18.39 ± 4.00 mV and a total solid content of 4.0 g/L Fe_3_O_4_@Al(OH)_3_ were produced as described by González-Gómez *et al*. [1]. In short, superparamagnetic Fe_3_O_4_@polyacrylic acid NPs were produced by co-precipitation, and submitted to forced chemical hydrolysis to produce Fe_3_O_4_@Al(OH)_3_ NPs, which were then magnetically separated, washed and re-dispersed in Milli-Q water.

mMSCs transduced with a lentiviral vector encoding for fLuc and enhanced green fluorescent protein were cultured in high-glucose Dulbecco’s Modified Eagle’s Medium (DMEM) containing GlutaMax supplemented with 15% fetal bovine serum and 1% penicillin-streptomycin (Gibco, ThermoFisher Scientific, Erembodegem, Belgium). Cells were grown in a humidified incubator at 37°C, 21% O_2_ and 5% CO_2_ (Binder, Tuttlingen, Germany).

Labeling of the mMSCs was performed as described by González-Gómez *et al.* [1]. In brief, 24h after plating 3 x 10^5^ cells, they were incubated with NPs (diluted in saline, 0.38 mM iron; non-RL (cold) or RL with 10 MBq of [^18^F]NaF) for 1h. Afterwards, the cells were washed and detached from the plate using TrypLE (Gibco). A Neubauer cell counting chamber (Hirschmann, Eberstadt, Germany) was used to count the cells and 1 x 10^5^ were resuspended in saline for IV injection (mMSC + (RL) NPs).

***In vivo follow-up of biodistribution of Fe_3_O_4_@Al(OH)_3_ NPs and NP-labeled mMSCs***

*PET/MRI scanner:* A BioSpec 70/30 small animal MRI scanner with a horizontal bore of 30 cm (Bruker Biospin, Ettlingen, Germany) in combination with a quadrature radio-frequency resonator (transmit/ receive; inner diameter 4.0 cm; Bruker Biospin) was used for image acquisition. The small animal MR scanner is equipped with actively shielded gradients (200 mT m^-1^) and a PET insert (3 rings of monolithic LYSO crystals coupled to silicon photomultipliers (Bruker Si-PET insert); Bruker BioSpin) [2]. The latter has a resolution of 0.9 – 1 mm, sensitivity of 11% and a field of view of 80 mm x 80 mm x 150 mm. During all acquisitions, the breathing rate and body temperature of the mice were monitored using a physiological monitoring system (Small Animal Instruments Inc., Stony Brook, NY, US) and were maintained at 60-80 min^-1^ and 37 ± 1 ˚C, respectively.

*Computed tomography (CT):* To evaluate the bone uptake of free [^18^F]F^-^, the mouse bed was moved to a dedicated small animal CT scanner after acquisition of the PET/MRI scans for a subgroup of animals (injected with saline, [^18^F]NaF, RL NPs or mMSC + RL NPs). A CT scan with an isotropic resolution of 51.42 µm was acquired using the following parameters: 50kVp X-ray source, 1 mm aluminum filter, 918 µA current, 55 ms exposure time per projection, 5 projections per positions with increments of 0.9° and a total angle of 180° (scan time of approximately four min).

*Long-term follow-up:* Long-term follow-up of cold NPs and NP-labeled mMSCs was performed using BLI and MRI. T_2_ maps and 3D T_2_-weighted MRI were acquired before, four hours after and, one day, two and seven days post injection (PI; for scan parameters see Table 1).

BLI was performed one hour, eight hours and one and two days PI. 126 mg/kg D-luciferin (Promega Benelux, Leiden, the Netherlands) was injected subcutaneously and light was captured using an IVIS Spectrum camera (Perkin Elmer, Waltham, MA, USA). Following parameters were used: luminescent exposure of 60 s, F/ stop = 1, field of view = 22.8 cm, subject height = 1.5 cm and binning factor = 8. Images of the mice in supine position were acquired until a plateau value for total light flux was reached.

***Image analysis***

*MRI*

T_2_ parametric maps were generated using the manufacturer’s Paravision 6.0.1. software (Bruker Biospin). T_2_ values of the liver were determined in ImageJ (NIH, Bethesda, MD, USA) by drawing an 8 mm² circular region of interest (ROI) on 3 adjacent slices and averaging the values. The size of the ROI was kept constant across all slices and animals.

An in-house written tool (Python version 2.7 in Spyder) was used to average, spatially smooth (0.75 voxels) and temporally smooth (rolling mean with window level of 5) the DCE images. The 3D T_2_ weighted anatomical MRI scans were resized to match the geometry of the DCE-MRI and were used to manually delineate the liver in 3D Slicer version 4.8.1 (https://www.slicer.org; [3]). Afterwards, the volume of interest (VOI) was transferred to the DCE scan and the mean signal intensity within the liver on each frame was determined using the Multi Volume Explorer tool (3D Slicer).

*CT*

CT scans were reconstructed using Nrecon v1.16.10.4. software (Bruker CT) according to the following parameters: Gaussian smoothing of 1%, reduction of ring artefacts of 3%, beam hardening of 10% and a manual determination of the post alignment (values between -1.5 and 0.5). Next, a binary bone image was created in CTAn v1.16.3.0. (Bruker CT). The signal from MR monitoring probes and liver calcifications was manually removed in ITK-SNAP [4]. The binary image was then multiplied with the original image to create a CT-based bone mask. Lastly, the image resolution was decreased to 102.84 µm isotropic to reduce physical scan size and allow easier handling.

*PET*

All one-hour static scans were reconstructed using a maximum likelihood estimation method (MLEM; parameters: 12 iterations, 0.5 mm isotropic resolution, decay/ scatter/ random correction). Furthermore, they were reframed into 20 frames of one minute and eight frames of five minutes to study the biodistribution of the NPs/cells over time. Afterwards, a dynamic reconstruction of the frames was performed using the same MLEM algorithm.

Reconstructed PET images were analyzed in PMOD, version 3.9 (PMOD Technologies, Zürich, Switzerland). Standardized uptake value (SUV) normalization was performed according to the following formula:

$SUV = Activity concentration in organ/\left[ \frac{injected activity}{weight animal} \right]$ (1)

PET images were then smoothed (0.5 mm Gaussian smoothing) and manually overlaid with the 3D T_2_-weighted MRI or the reduced resolution, CT-based bone mask within the PMOD FuseIT tool. To determine the mean SUV in liver, lungs, spleen, bladder and kidneys, VOIs were drawn around these organs on the 3D T_2_-weighted MRI and transferred to the SUV normalized PET images. Average ^18^F uptake in the bone was determined by drawing an automatic isocontour around the bone mask and quantifying the PET signal within the VOI. Due to low uptake of the RL NPs/cells in the heart, the left ventricle was only visible on the dynamically reframed scans right after injection (second frame of the dynamic PET reconstruction corresponding to the blood pool). Therefore, the left ventricle input function was determined by placing a 1 mm radius VOI within the structure of interest on the second frame of the dynamically reframed PET scan, which was extrapolated to all other frames.

***Leukocytosis***

The effect of cold NP or NP-labeled mMSCs on leukocytosis was evaluated after IV injection of either compound. Similarly, saline was injected in a control group. Blood was taken from the mice on day one and day seven after injection of the experimental compound and collected in Eppendorf tubes, which were washed with UltraPure 0.5 M EDTA (pH = 8; Gibco) to prevent the blood from clotting. Analysis of the number and differentiation of white blood cells (WBC) was performed using the ABX Micros 60 (Horiba, Lier, Belgium).

**References**

1. González-Gómez MA, Belderbos S, Yañez-Vilar S, Piñeiro Y, Cleeren F, Bormans G, Deroose CM, Gsell W, Himmelreich U, Rivas J (2019) Development of Superparamagnetic Nanoparticles Coated with Polyacrylic Acid and Aluminum Hydroxide as an Efficient Contrast Agent for Multimodal Imaging. Nanomaterials 9:1626

2. Gsell W, Molinos C, Correcher C, Junge S, Heidenberg M, Himmelreich U, Deroose CM, Gonzalez AJ (2019) Characterization of preclinical PET insert for 7T: beyond NEMA testing. Proc. Intl. Soc. Mag. Reson. Med. 27

3. Fedorov A, Beichel R, Kalpathy-Cramer J, et al (2012) 3D Slicer as an image computing platform for the Quantitative Imaging Network. Magn Reson Imaging 30:1323–1341

4. Yushkevich PA, Piven J, Hazlett HC, Smith RG, Ho S, Gee JC, Gerig G (2006) User-guided 3D active contour segmentation of anatomical structures: significantly improved efficiency and reliability. Neuroimage 31:1116–28

**Supplementary Figures**

**
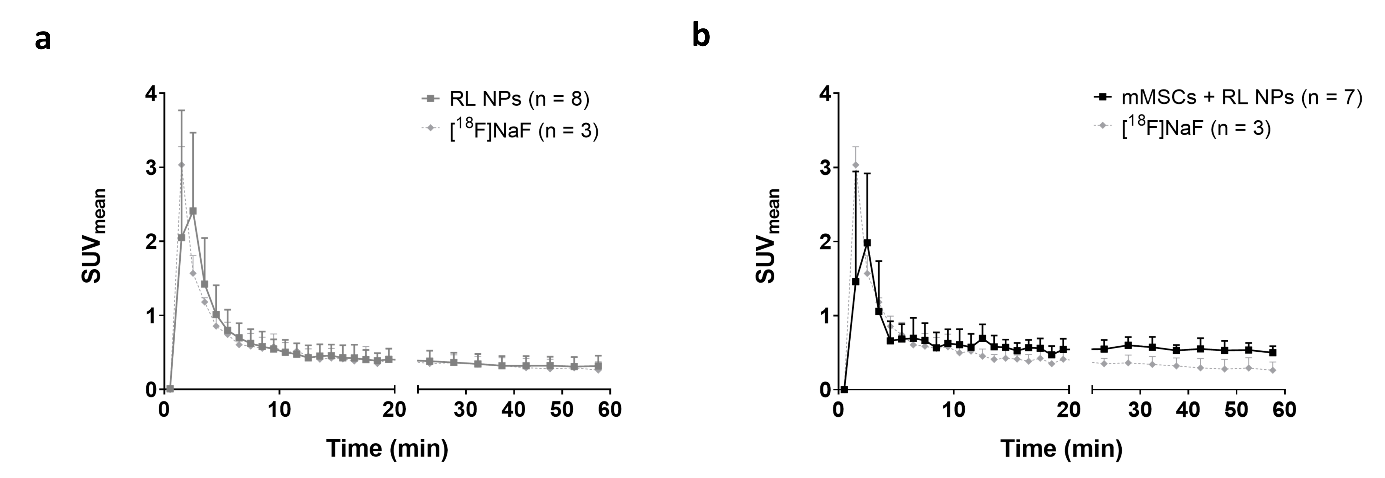
**

**Fig. S1 Image-derived left ventricle input functions.** After reframing of the static scans into 20 one-minute frames and 8 five-minute frames, the left ventricle input function of mice injected with **a** ^18^F-labeled Fe_3_O_4_@Al(OH)_3_ NPs (radiolabeled or RL NPs) or **b** mMSCs labeled with these NPs (mMSCs + RL NPs) were determined, and compared with those of a control group injected with free radiotracer. Similar left ventricle input functions were measured in all three conditions as seen by the mean standardized uptake values (SUV_mean_) with only a significant difference between RL NPs and [^18^F]NaF-injected mice (*p* < 0.05) and mMSCs + RL NPs and [^18^F]NaF-injected animals (*p* < 0.001) immediately after injection, respectively (based on a repeated measures two-way ANOVA with Bonferroni correction).


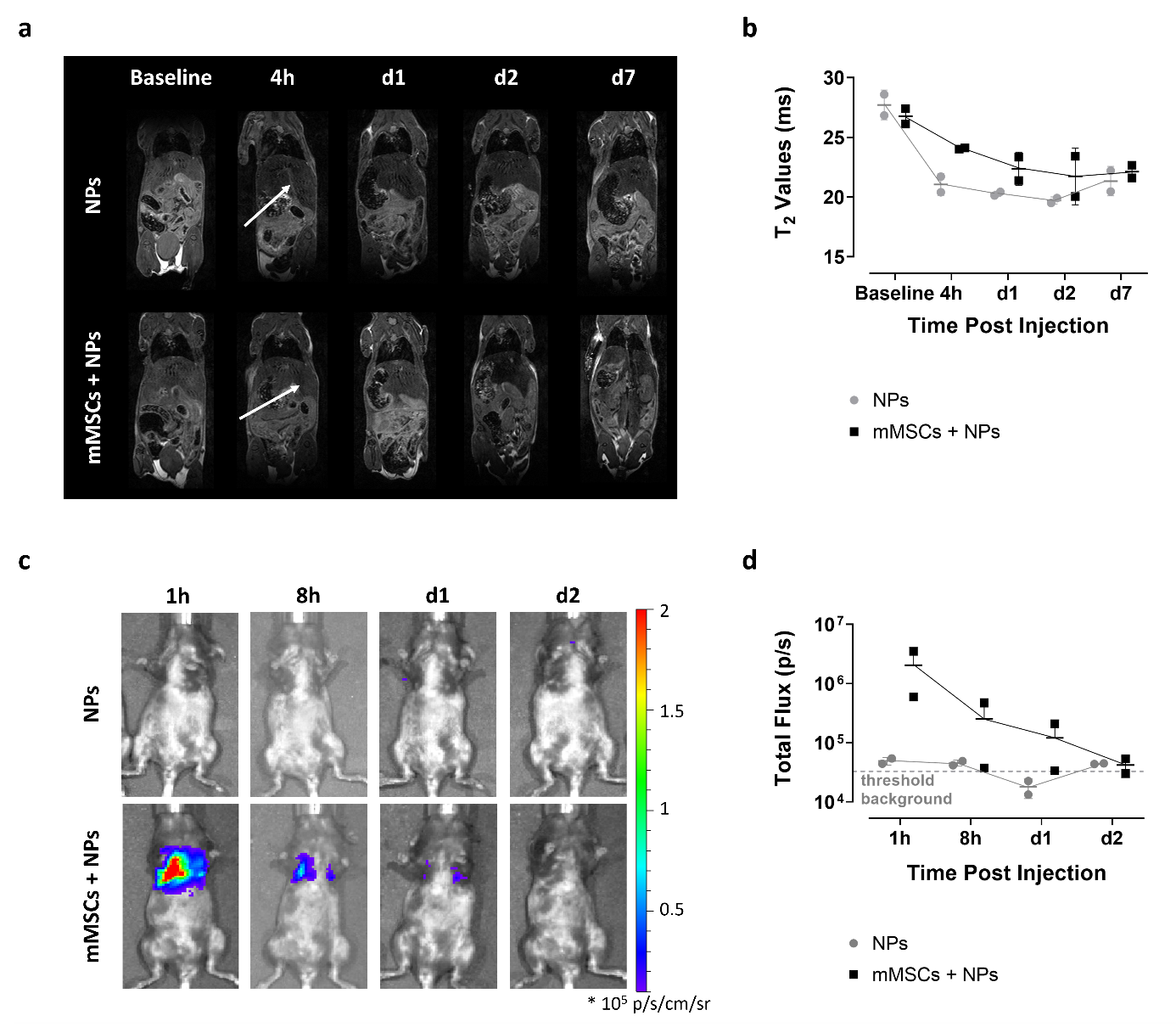


**Fig. S2 Long-term follow-up of Fe_3_O_4_@Al(OH)_3_ NPs and mMSCs + NPs biodistribution.** Bioluminescence images, 3D T_2_-weighted anatomical MRI and T_2_ maps were acquired to study the long-term biodistribution of ^18^F-labeled Fe_3_O_4_@Al(OH)_3_ NPs (RL NPs) and mMSCs labeled with RL NPs (mMSCs + RL NPs). **a** Hypointense liver signals (indicated by white arrows) on the 3D T_2_-weighted anatomical MRI images of both experimental groups indicate the uptake of the NPs. **b** Lower T_2_ values of the liver 4h post injection confirm the anatomical images. The decrease after injection of the NPs is larger when compared to the cells. In contrast, after seven days, the values either stagnate or increase slightly again. **c** After the injection of D-luciferin, a clear bioluminescence signal from the lungs was seen after injection of mMSCs + NPs, which was present until 24h post injection. **d** These results were further confirmed by the quantification of total flux confirm present within the lungs. However, a large inter-group variability could be seen. Threshold background represents the average total flux within the lungs of mice injected with NPs (control group for BLI). No statistical analysis was performed as *n = 2*/ group.**
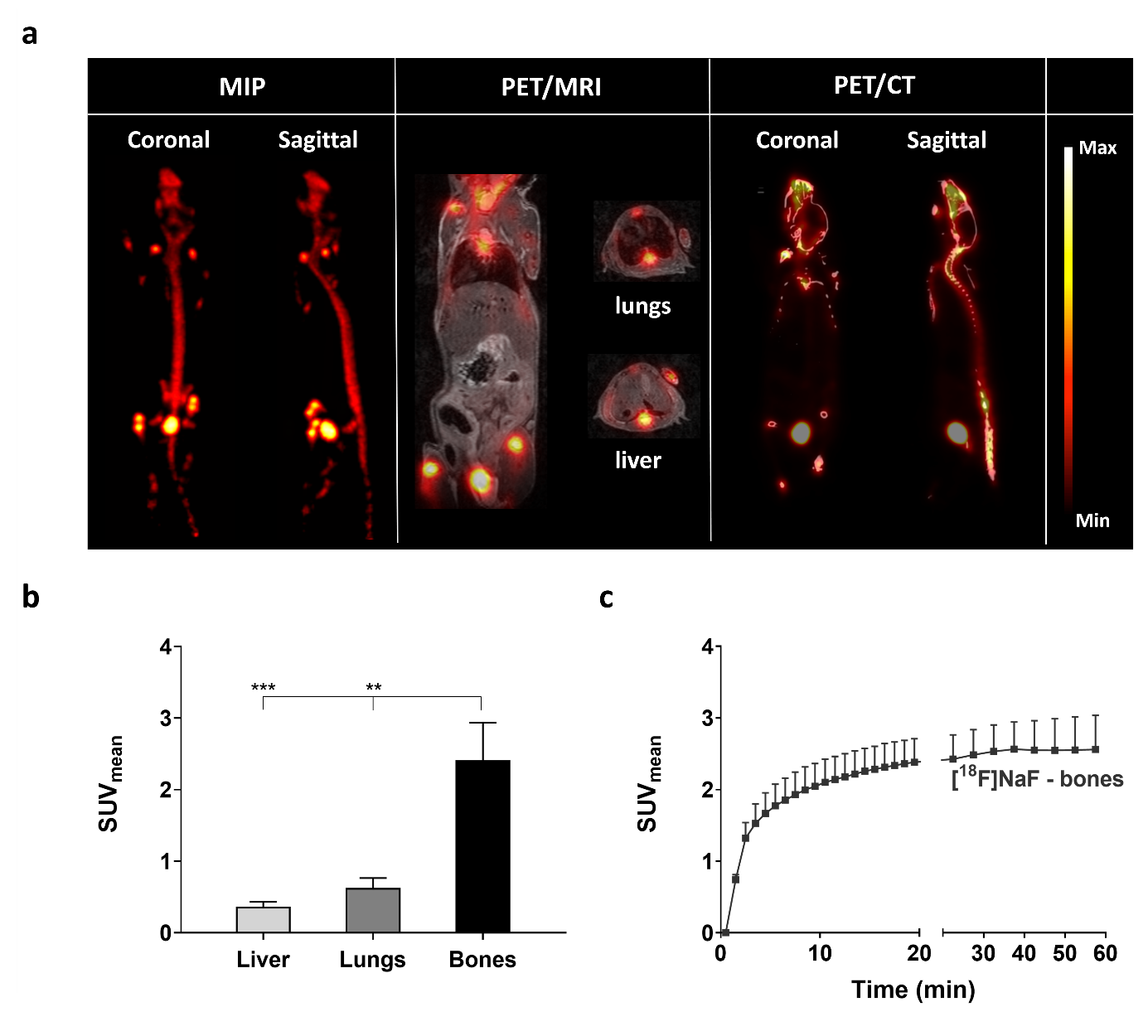
**

**Fig. S3 PET/MRI in combination with PET/CT after injection of [^18^F]NaF.** [^18^F]NaF was injected in a control group to compare to the bone uptake in both experimental groups, and radiotracer biodistribution was studied in different organs. **a** While a clear signal from the bones and the bladder is visible on the maximum intensity projection (MIP) images, and the static ^18^F-PET (SUV = 0–9) and 3D T_2_-weighted anatomical or whole-body CT overlay (*n = 3*), no tracer uptake in lungs or liver was seen, **b** which was also confirmed by the quantification of the average SUV (SUV_mean_; one-way ANOVA with Bonferroni multiple comparisons test, *** p* = 0.0014 and **** p* = 0.006). **c** Dynamic quantification of the signal in the bones indicates a relatively quick and stable uptake of the radiotracer in the skeleton after injection.


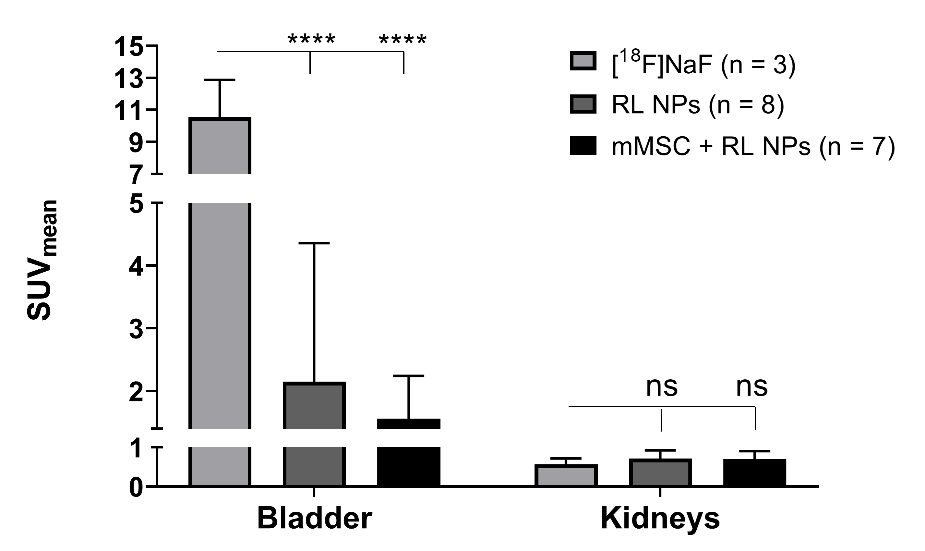


**Fig.** **S4** **Renal and bladder secretion of ^18^F**. After injection of [^18^F]NaF, ^18^F-labeled Fe_3_O_4_@Al(OH)_3_ NPs (RL NPs) or RL NP-labeled mMSCs (mMSC + NPs) biodistribution, the secretion of the radiotracer via the bladder and kidneys was evaluated. While a significantly higher standardized uptake value (SUV_mean_) was seen after injection of [^18^F]NaF compared to the two other groups, no difference in tracer uptake in the kidneys was measured (two-way ANOVA with Bonferroni correction for multiple comparisons; ***** p* < 0.0001 and ns = non-significant).


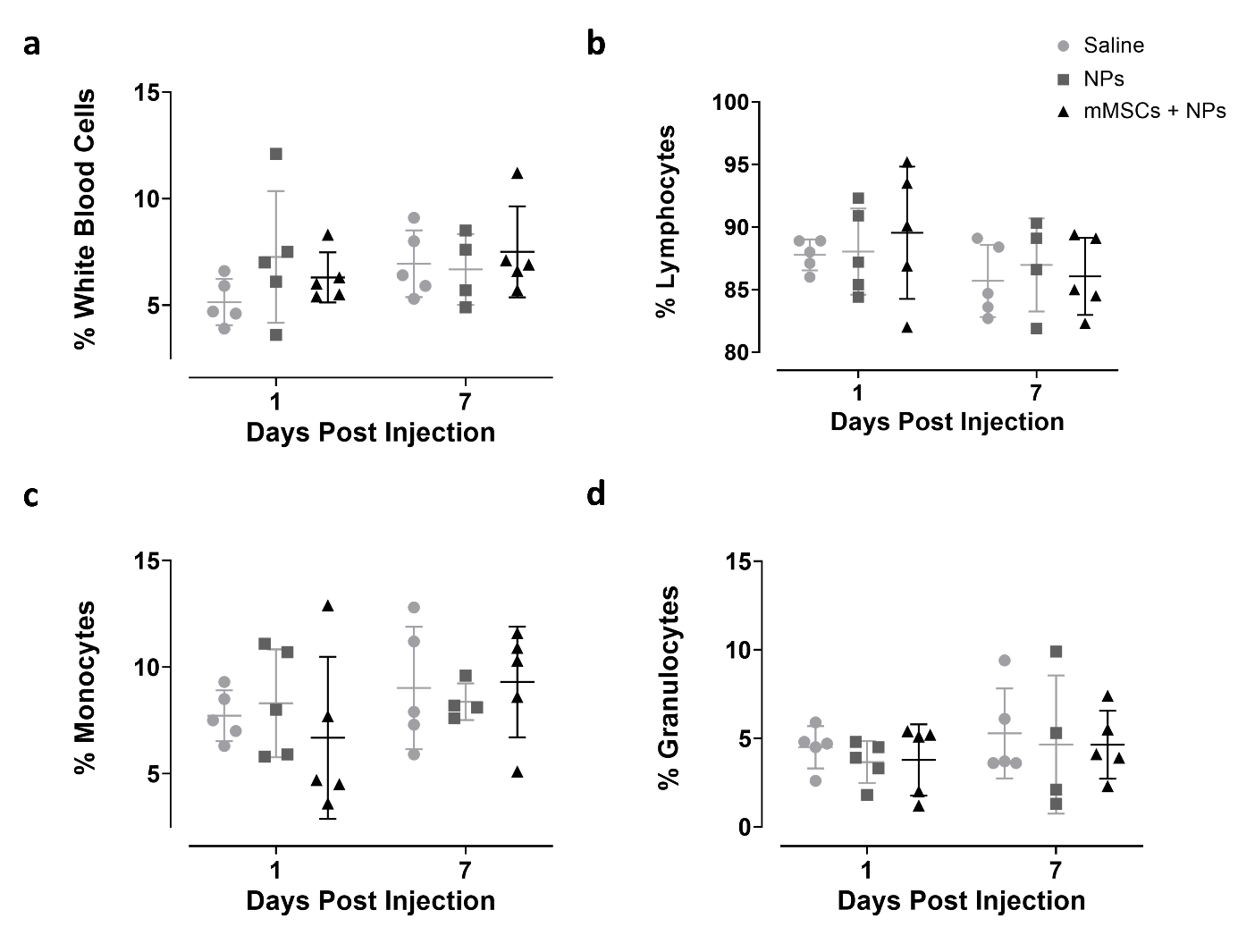


**Fig. S5**. **Effect on white blood cells after injection of Fe_3_O_4_@Al(OH)_3_ NPs or NP-labeled mMSCs. a** No effect was seen on the percentage of white blood cells within the blood of mice injected with saline (control), Fe_3_O_4_@Al(OH)_3_ NPs or mMSCs labeled with these NPs (mMSCs + NPs) (*n = 5*/group). **b - d** Furthermore, within the differentiated white blood cells: **b** lymphocytes, **c** monocytes and **d** granulocytes; no time-related or compound-related differences were found (mixed-effects model, Bonferroni multiple comparisons test).
